# The sustained effect of 5-week EmotionCore mindfulness training on emotion regulation and emotional intelligence: heterogeneous benefits for depression and anxiety across subgroups

**DOI:** 10.3389/fpsyt.2025.1622626

**Published:** 2025-07-18

**Authors:** Hui Kou, Wei Luo, Yiwei Wang, Jia Wu, Xiaodong Li, Yi Wu, Qianguo Xiao, Taiyong Bi

**Affiliations:** ^1^ Research Center of Humanities and Medicine, Zunyi Medical University, Zunyi, China; ^2^ The Institute of Ethnology and Anthropology, Chinese Academy of Social Sciences, Beijing, China; ^3^ Art School, Changsha Normal College for Preschool Education, Changsha, China

**Keywords:** EmotionCore mindfulness training, emotion regulation, emotional intelligence, mediation analysis, latent profile analysis

## Abstract

**Background:**

The present study investigated the sustained effects of a 5-week EmotionCore mindfulness training program on emotion regulation, emotional intelligence (EI), trait mindfulness, depression, and anxiety among 120 undergraduates, while exploring the mediating mechanisms of training effect and heterogeneous effects across subgroups.

**Methods:**

Participants were randomly assigned to a mindfulness group (n=60) or a waitlist control group (n=60), with assessments conducted at baseline (T1), post-training (T2), and one-month follow-up (T3).

**Results:**

Results demonstrated that mindfulness training significantly enhanced adaptive cognitive emotion regulation strategies (ACERSs), EI, and trait mindfulness at both T2 and T3. Both cross-section and longitudinal mediation models revealed that improvements in ACERSs and EI fully and sequentially mediated the relationship between mindfulness training and trait mindfulness enhancement. Latent Profile Analysis revealed that the high-risk group (high baseline anxiety/depression) exhibited greater reductions in anxiety and depression at T2 and T3 compared to the low-risk group.

**Conclusion:**

These findings suggest EmotionCore mindfulness training fosters trait mindfulness through improvements in ACERSs and EI, and its targeted efficacy is specifically notable within high-risk populations.

## Introduction

Mindfulness is a state characterized by intentionally directing attention to present-moment experiences - both internal and external - with a curious, open, receptive, and non-judgmental attitude (1, 2). Mindfulness is not about fighting or controlling thoughts, but rather cultivating an intentional and non-judgmental awareness of the present moment—liberating oneself from the entanglement of ruminating over past experiences and the relentless anxieties of the future (2). Trait/dispositional mindfulness refers to an individual’s characteristic tendency to maintain awareness of the present moment in a nonreactive and nonjudgmental manner (3). Previous studies revealed that mindfulness training is associated with increased trait mindfulness (4–7). Recently, mindfulness training has been applied in various settings, including clinical psychology, education, and organizational contexts, yielding promising results. Mindfulness training enhances meta-awareness by consciously observing and monitoring thoughts, emotions, and behaviors, empowering individuals to disrupt maladaptively automatic cognition and reaction patterns (8–10). A large number of studies have demonstrated that mindfulness training can improve mental health and well-being (11–17), and reduce emotional problems such as anxiety and depression (12, 15–23). In the present study, we developed a program of EmotionCore mindfulness training, to specifically enhance emotional perception and regulation. First, the efficacy of this specialized intervention in improving trait mindfulness was verified. Then, we examined its effect on alleviating anxiety and depression, while evaluating the long-term sustainability of these training benefits, as well as the precise mechanisms underlying these training effects. Existing evidence supports that mindfulness-based interventions often exhibit protective effects over long periods, such as 1-month follow-up (24, 25), 1.5-month follow-up (26), 4-month follow-up (27), 6-month follow-up (28, 29), 12-month follow-up (29), 2.5-year follow-up (30), and even 4-year follow-up (31). Meta-analyses revealed that the effect of mindfulness training may be retained for a period of one to twelve months (32, 33). In the present study, we evaluated the training effect after a one-month break. This period was selected to balance the assessment of immediate post-intervention outcomes with the evaluation of early sustainability, while simultaneously reducing participant attrition rates. We hypothesized that the effect of mindfulness training may retain at the one-month follow-up.

Theories propose that mindfulness training is an effective way to achieve positive psychological outcomes through decentering, disrupting automatic maladaptive cognitive patterns that may be closely associated with emotion regulation. For example, according to the mindful coping model, mindfulness fosters a “decentered” metacognitive state that enhances cognitive flexibility, thereby disrupting maladaptively automatic reactivity and activating adaptive responses (34, 35). Meanwhile, the mindfulness-to-meaning theory further posits that mindfulness can neutralize initial cognitive appraisals for stressors by disrupting the automatic activation of habitual cognition patterns to liberate attention from rumination on stressors, while fostering more flexible and adaptive cognitive processes that encourage positive psychological outcomes (36). Furthermore, the RICH model of mindfulness explicitly delineates four core mechanisms, that is relaxation, insight, contact, and harmony, through which mindfulness exerts its beneficial effects on a number of lower-level intermediate factors, including emotion regulation like rumination and reappraisal, etc., ultimately enhancing psychological well-being (37). Previous studies have demonstrated that emotion regulation (e.g., the ability to manage negative emotions and rumination) plays mediating roles in the relationship between mindfulness and mental health (38–41), in the training effects on internalizing symptoms and perceived stress (42), and in the training effects on psychological well-being (43). Among emotion regulation, cognitive emotion regulation (CER) involves the use of a range of conscious cognitive processes that can regulate emotional response, which can be categorized as either adaptive (e.g., acceptance, refocus on planning, positive refocusing, positive reappraisal, and putting into perspective) or nonadaptive (e.g., self-blame, blaming others, rumination or focus on thought, and catastrophizing) strategies (44). Mindfulness is associated with the utilization of cognitive emotion regulation strategies (CERSs). Previous studies revealed that mindfulness was negatively correlated with nonadaptive CERSs as well as positively correlated with adaptive CERSs (45, 46), and both adaptive and nonadaptive CERSs played mediated roles in the relationship between mindfulness and perceived stress (47, 48). More direct evidence revealed that mindfulness training could improve the utilization of adaptive CERSs while reducing the utilization of nonadaptive CERSs (49), such as enhancing cognitive reappraisal and reducing rumination (50–52). Thus, we examined the effect of EmotionCore mindfulness training on the utilization of CERSs in the present study. Crucially, we incorporate a longitudinal design with multiple follow-up assessments conducted immediately and 1-month later to examine the stability and persistence of training effects. By employing mediated model analyses, we further investigated how CERSs enhancement mediated the training outcomes.

Emotion regulation is theoretically associated with Emotional Intelligence (EI), with both concepts sharing deep conceptual overlaps in their psychological constructs. EI refers to the ability to perceive and express emotions, integrate emotions into thoughts, understand and utilize emotions for reasoning, as well as regulate one’s own and others’ emotions (53, 54). The Four-Branch Model divides EI into four components: 1) Perceiving emotions, involving recognizing one’s own and other’s emotions, and expressing related needs; 2) Utilizing emotions to facilitate thinking, which refers to the ability to consciously utilize emotions to enhance cognitive processes and problem-solving; 3) Understanding emotions, which encompasses the capacity to understand and deduce the reasons behind emotional shifts within oneself or others and the significance conveyed by emotions; 4) Managing emotions, which refers to the capability to actively regulate their own or others’ emotions (55, 56). Within this framework, emotion regulation seems to emerge as a pivotal component of EI, emphasizing the effective and flexible application of emotion regulation strategies. Empirical studies revealed that EI could positively predict CERSs like reappraisal (57–59), and correlated with or predicted reduced emotion regulation difficulties (60, 61). Trait EI facilitated adaptive emotion regulation strategy selection (62). It could positively predict reappraisal (63), and negatively predict the frequency of use of typical dysfunctional emotion regulation strategies (64). Direct evidence revealed that EI training significantly influenced CER (65) and positive reappraisal (66). However, some researchers found that CERS could predict trait EI (67). Collectively, both emotion regulation and EI are involved in the multidimensional process of emotion management, and the flexible utilization and selection of emotion regulation strategies may substantially affect one’s capacity to effectively manage emotions across various contexts. Additionally, mindfulness has been found to be closely associated with EI (68–70); and mindfulness training could increase the level of EI (71–73). Therefore, it’s plausible to hypothesize that the EmotionCore mindfulness training first improved the utilization of emotion regulation strategies, subsequently enhancing EI.

According to the Whole Trait Theory (74), traits are divided into explanatory and descriptive parts, which are causally related. The descriptive side of traits is characterized by density distributions of states. Therefore, fluctuations in states may influence traits. Meanwhile, the explanatory part consists of mechanisms that produce traits, including cognition; and social-cognitive processes can explain density distributions of states (74). Therefore, it is reasonable to assume that the developmental pathway of mindfulness training involves cognitive shifts related to the emotions preceding, thereby consolidating the trait mindfulness. We thus hypothesize that the 5-week EmotionCore mindfulness training initially improves adaptive CERSs and EI, and subsequently enhances trait mindfulness.

Although mindfulness training is shown to enhance emotion regulation and EI, the associations between these enhancements and their roles in the training effect on mindfulness remain unclear. In the present study, we first examined the impact of 5-week EmotionCore mindfulness training on the utilization of CERSs, EI, trait mindfulness, anxiety, and depression. Then, we further examined whether the improvements of CERSs and EI played sequential mediating roles in the relationship between mindfulness training and trait mindfulness enhancement. At last, subgroups with different characteristics were identified to examine the targeted effects of EmotionCore mindfulness training through latent profile analysis. The present design advances our knowledge of mindfulness training in three aspects. First, the self-designed “EmotionCore mindfulness training” which targets emotional perception and regulation differs from traditional mindfulness training which often emphasizes generalized attention cultivation, providing a more targeted intervention protocol for emotion-related problems. Second, we adopt a longitudinal mediating model to infer the causal mechanisms underlying mindfulness training, elucidating the hierarchical change process in mindfulness-based interventions. Third, we applied the latent profile model to a group which is previously considered homogeneous, and revealed the heterogeneity in training sensitivity, providing empirical support for targeted interventions.

## Methods

### Participants

Power analysis indicated that a total sample size of 102 participants was needed to detect a significant difference in the training effect between groups (α=0.05, effect size=0.5, power= 0.8). A total of 120 undergraduates were recruited via online advertisements and randomly assigned to either the mindfulness group, which underwent an intensive 5-week mindfulness training program designed for emotional regulation, or the waitlist control group, which participated in two introductory lectures on mindfulness concepts. No significant age differences were observed between the mindfulness group (*M*=20.27, *SD*=1.30, ranging from 18 to 24) and the control group (*M*=19.97, *SD*=0.88, ranging from 19 to 24) (*t*(118) =1.48, *p*=0.142). No significant sex-based differences were observed between the mindfulness group (50 females) and the control group (42 females) *(χ²*=2.98, *df*=1, *p*=0.084).

Inclusion criteria: Undergraduate students aged 18–24, fluent in Chinese, with normal or corrected-to-normal vision.

Exclusion criteria: Self-reported history of neurological/psychiatric disorders; self-reported anxiety/depressive disorders; current use of psychotropic medications; prior experience with formal mindfulness training or meditation practices.

### Ethics approval statement

All procedures conducted in this study involving human participants are approved by the Ethical Committee of Human Research at Zunyi Medical University. Informed consent was obtained from all participants prior to the experiment. After completing the experiment, control group members were also offered mindfulness training if they volunteered to participate.

### Measurements

*The Five Facet Mindfulness Questionnaire (FFMQ)* It was employed to assess participants’ mindfulness levels, developed by Baer, Smith, Hopkins, Krietemeyer and Toney (75). This 39-item questionnaire comprises five subscales: Observing (e.g., “When I take a shower or bath, I stay alert to the sensations of water on my body”), Describing (e.g., “I can easily put my beliefs, opinions, and expectations into words”), Acting with awareness (e.g., “When I do things, my mind wanders off and I’m easily distracted”), Non-judging (e.g., “I tell myself I shouldn’t be feeling the way I’m feeling”) and Non-reacting (e.g., “When I have distressing thoughts or images, I just notice them and let them go”). Participants rated each item on a 5-point Likert scale ranging from *1=very rarely true* to *5=always true*. The Chinese version of the FFMQ demonstrated a Cronbach’s α coefficient of 0.70 in non-clinical populations (76). In the present study, the Cronbach’s α coefficient is 0.820.

*The Emotional Intelligence Scale (EIS)* It was developed by Schutte, Malouff, Hall, Haggerty, Cooper, Golden and Dornheim (77) to assess individuals’ capacity to perceive, understand, control, and manage emotion. This 33 items scale comprises four subscales: Appraisal of other’s emotion (e.g., “Other people find it easy to confide in me”), Appraisal and Expression of Own Emotion (e.g., “I know when to speak about my personal problems to others”), Regulation of Emotion (e.g., “When my mood changes, I see new possibilities”), and Utilization of Emotion (e.g., “Emotions are one of the things that make my life worth living”). Each item is rated on a 5-point Likert scale, ranging from *1=strongly disagree* to *5=strongly agree*. The EIS demonstrated a Cronbach’s α of 0.90 and a two-week test-retest reliability coefficient of 0.78 (77). In the present study, the Cronbach’s α coefficient is 0.852.

*The Cognitive Emotion Regulation Questionnaire (CERQ)* It was initially developed by Garnefski, Kraaij and Spinhoven (44) and was subsequently translated and revised into Chinese version by Zhu, Luo, Yao, P.Auerbach and JohnR.Z.Abela (78). The questionnaire consists of 36 items and nine subscales. These subscales evaluate a range of strategies employed in cognitive emotion regulation, including self-blame, blaming others, acceptance, refocus on planning, positive refocusing, rumination or focus on thought, positive reappraisal, putting into perspective, and catastrophizing. Each item is rated on a 5-point Likert scale ranging from *1=strongly disagree* to *5=strongly agree*, with higher subscale scores reflecting more frequent use of that specific strategy during negative experiences. The adaptive cognitive emotion regulation strategies (CERSs) include acceptance, putting into perspective, refocusing on planning, positive refocusing, and positive reappraisal subscales. The nonadaptive CERSs consist of self-blame, blaming others, rumination or focus on thought, and catastrophizing subscales. In the Chinese population, the subscale reliability ranged from 0.48 to 0.89 (78). In the present study, the Cronbach’s α coefficient is 0.896.

*The Emotion Regulation Questionnaire (ERQ)* This 10-item measure was developed by Gross and John (79). It covers two factors: Cognitive Reappraisal (e.g., I control my emotions by changing the way I think about the situation I’m in) and Expressive Suppression (e.g., I control my emotions by not expressing them). Each item is rated on a 7-point Likert scale from *1=strongly disagree* to *7=strongly agree*. The Cronbach’s α coefficient was 0.79 for Cognitive Reappraisal and 0.73 for Expressive Suppression, and the test-retest reliability across 3 months was 0.69 for both scales (79). In the present study, the Cronbach’s α coefficient is 0.711.

*Self-Rating Depression Scale (SDS)* (80) This 20-item measurement is designed to assess the frequency of depressive symptoms experienced over the past seven days. Each item is rated on a 4-point Likert scale from *1 =none or a little of the time* to *4= most or all of the time*. The cumulative score reflects symptom severity. In the present study, the Cronbach’s α coefficient is 0.638.

*Self-Rating Anxiety Scale (SAS)* (81) This 20-item measurement is used to assess the frequency of anxious symptoms experienced over the past seven days. Each item is rated on a 4-point Likert scale from *1 =none or a little of the time* to *4= most or all of the time*. The cumulative score reflects anxiety severity. In the present study, the Cronbach’s α coefficient is 0.680.

### Procedure

Participants first completed the baseline measurement (T1), filling in all the questionnaires.

Following the baseline assessment, participants were randomly assigned to either the training group, which underwent mindfulness training sessions, or the control group, which participated in two introductory lectures on mindfulness concepts. Participants in the training group engaged systematically in a five-week mindfulness-based stress reduction (MBSR) program. The training was conducted in group-based sessions comprising 30 participants per group, guided by a therapist with three years of expertise in MBSR instruction and sustained meditation practice. The EmotionCore curriculum was designed based on Kabat-Zinn’s foundational MBSR framework (2) and mindfulness-based cognitive therapy (MBCT) (82), and focused on emotional perception and regulation. From MBSR, we adopted foundational practices like mindful eating (to enhance present-moment sensory awareness) and body scans (to cultivate non-judgmental bodily awareness), which form the basis for emotional perception. From MBCT, We integrated emotion-cognition link exercises (e.g., exploring how thoughts shape emotional responses) and non-reactivity training (to reduce automatic emotional reactions), aligning with MBCT’s focus on breaking maladaptive cognitive-emotional cycles. Each week set an emotion-centered theme through a two-hour session, with detailed training content outlined in **Table 1**. Unlike MBSR (which targets general stress reduction) or MBCT (primarily for depression relapse prevention), EmotionCore is explicitly emotion-centric. Its weekly themes (e.g., “mindfulness and emotional recognition,” “mindfulness and emotion regulation”) systematically train participants to identify emotional components (cognitive appraisal, physiological arousal, behavioral responses; Week 2), understand emotion-cognition dynamics (Week 3), identify the experience and body responses to emotion (Week 4), and apply flexible regulation strategies (Week 5). This structure differentiates EmotionCore by prioritizing emotional perception and adaptive regulation, making it more suitable for populations with subclinical emotional distress (e.g., college students). After the training, participants undertook daily mindfulness exercises at home, and reported their feelings to the trainer. All participants allocated to the mindfulness training group successfully completed the five scheduled training sessions.

**Table 1:** The contents of weekly mindfulness training.

Sessions	Topics	Course contents	Daily homework
Preparation	Course introduction	An introduction to the content, format, duration, and rules of the mindfulness training course, encompassing respect, confidentiality, non-judgment, and other principles.	
First week	The first experience with mindfulness	Understanding mindfulness through mindful eating of raisins and a mindfulness body scan.	15-minute mindful breathing or body scan (guided via audio recordings).
Second week	Mindfulness and emotional recognition	Recognizing emotions (cognitive evaluation, physiological arousal, subjective feelings, behavioral manifestations and responses) - recognition, mindful awareness, and experience.	10-minute emotion observation practice (e.g., observing emotional feelings, cognitive evaluation, physiological arousal, and behavioral responses without judgment).
Third week	Mindfulness and emotional cognition	Understanding the relationships between emotional experience and cognition and reactions, learning the attitude of allowing and letting go.	10-minute emotion understanding practice (e.g., observing the relationships between emotion experience and cognition and reactions without judgment).
Fourth week	Mindfulness and emotional response	Identifying the experience and body responses to emotion, learning the attitude of non-reactivity.	10-minute emotional response identification practice (e.g., adopting the attitude of non-reactivity towards a recent emotional event).
Fifth week	Mindfulness and emotion regulation	Flexible mindfulness response emotional process and course summary	10-minute flexible regulation practice (e.g., reappraising a recent emotional event).

Immediately following the training session, participants underwent post-training assessments (T2) same as the baseline measurements. These identical measurements were repeated one month post-training (T3) to track longitudinal outcomes.

Once all measurements were concluded, participants in the control group were also offered the opportunity to participate in mindfulness training if they wished.

### Statistical analysis

First, we examined the differences in scale scores between groups at T1 with a MANOVA, to test if there were any group biases at the baseline level. Next, independent-sample t-tests were conducted on the improvement of measures (i.e., T2-T1 or T3-T1), with the group serving as the independent variable.

Second, cross-sectional mediation models were constructed based on previous studies (83–85), with mindfulness training (0=control group, 1=mindfulness group) as the predictive variables, the improvements of adaptive CERSs and EI at T2/T3 as the mediators, and the improvements of trait mindfulness at T2/T3 as outcome variables. Furthermore, longitudinal mediation effects could be revealed in longitudinal designs. Multiple longitudinal mediation models have been proposed for mediation analysis, including the cross-lagged panel mediation models (86), the latent growth mediation models (87, 88), and the latent change score mediation models (89). The cross-lagged panel model can be used with at least three waves of measurement to achieve a fully longitudinal mediation model. This methodological approach defines mediation as a longitudinal process considering embedded causal sequences: a hypothesized predictor is measured prior to the hypothesized mediator, and the hypothesized mediator is measured prior to the hypothesized outcome variable (86). Therefore, a cross-lagged mediation model was based on the latent change scores, with mindfulness training (0=control group, 1=mindfulness group) serving as the independent variable, the improvements of adaptive CERSs and EI at T2 as mediators, and the improvement of FFMQ at T3 as the dependent variable. The improvements were all transformed into standardized scores before entering the models. The PROCESS macro (Model 6) for SPSS provided by Preacher and Hayes (84) was used to test the mediating effects.

Finally, Latent Profile Analysis (LPA) was conducted by Mplus 8.3 using maximum likelihood estimation. LPA is a categorical latent variable approach designed to identify individuals from a heterogeneous population into homogenous subgroups, within a subgroup who often exhibit shared observable characteristics, by analyzing shared patterns in their responses to a defined set of observed variables (90, 91). In the present study, LPA aimed to identify heterogeneity in intervention response, i.e., differences in training effects among participants with varying baseline characteristics. Since the waitlist group received no training (only lectures), LPA in this group could not reflect the specific effects of the intervention. Therefore, LPA was conducted only within the mindfulness group. Eight continuous variables were included: FFMQ, EI, ERQ-CR (Cognitive Reappraisal), ERQ-ES (Expressive Suppression), SDS, SAS, adaptive CERSs, and nonadaptive CERSs at T1. To avoid local maxima, 500 random start values with 50 final stage optimizations were implemented to ensure proper convergence. The optimal class was determined through a comprehensive evaluation of fit indices including the Akaike Information Criterion (AIC), Bayesian Information Criterion (BIC), Sample-Size Adjusted BIC (aBIC), Lo-Mendell-Rubin adjusted likelihood ratio test (LMR-LRT), and bootstrap likelihood ratio test (BLRT), with Entropy values assessed for classification quality.

## Results

### Baseline level

We conducted a comprehensive MANOVA to examine scores across multiple psychological measures: FFMQ, EI, adaptive CERSs, nonadaptive CERSs, ERQ-CR, ERQ-ES, SAS, and SDS. As illustrated in **Supplementary Table S1**, the analysis revealed no significant differences between the mindfulness group and the control group (*Fs* < 2.45, *ps* > 0.120), indicating that the two groups were well-matched at the baseline level.

### Post-training and follow-up

The scores of all measurements at T2 and T3 were displayed in **Supplementary Table S2**.

We first calculated the improvements in measurements by subtracting the scores at T1 from the scores at T2 or T3 (**Table 2**). Independent-sample t-tests on the improvements of measurements revealed that the mindfulness group demonstrated greater improvements in trait mindfulness (T2: *t*(118)=-2.72, *p*=0.008; T3: *t*(118)=-2.08, *p*=0.039), EI (T2: *t*(118)=-2.22, *p*=0.029; T3: *t*(118)=-2.53, *p*=0.013), and adaptive CERSs (T2: *t*(118)=-3.47, *p*=0.001; T3: *t*(118)=-3.07, *p*=0.003) at both T2 and T3. The mindfulness group also demonstrated greater improvements in anxiety (*t*(118)=2.10, *p*=0.038) and expressive suppression (*t*(118)=2.39, *p*=0.019), but only at T2. However, there weren’t any significant improvements in depression, cognitive reappraisal, and nonadaptive CERSs at both T2 and T3 (all *ps*>0.05). These results indicate sustained improvements in trait mindfulness, EI, and adaptive CERSs.

**Table 2:** Between-group differences in the improvements of all measurements at T2 and T3: descriptive statistics and results of independent-sample t-test (*N*=120).

Measurements	Control group (*n*=60)		Mindfulness group (*n*=60)		t	p
	*M*	*SD*	*M*	*SD*		
ΔFFMQ_T2-T1_	-1.00	6.05	3.08	9.95	-2.72	**0.008**
ΔFFMQ_T3-T1_	-0.30	6.61	2.40	7.56	-2.08	**0.039**
ΔEI_T2-T1_	-1.92	10.53	2.17	9.64	-2.22	**0.029**
ΔEI_T3-T1_	-3.92	9.85	0.40	8.81	-2.53	**0.013**
ΔERQ_CR_T2-T1_	-0.07	3.28	0.62	4.55	-0.94	0.347
ΔERQ_CR_T3-T1_	-0.13	4.19	0.70	4.77	-1.02	0.311
ΔERQ_ES_T2-T1_	0.82	4.25	-0.93	3.76	2.39	**0.019**
ΔERQ_ES_T3-T1_	1.12	4.07	0.72	4.00	0.54	0.588
ΔSDS_T2-T1_	-1.35	8.00	-3.50	8.14	1.46	0.148
ΔSDS_T3-T1_	1.10	7.87	-1.50	9.01	1.69	0.094
ΔSAS_T2-T1_	1.19	7.28	-1.50	6.76	2.09	**0.038**
ΔSAS_T3-T1_	3.42	9.24	2.48	8.53	0.58	0.565
ΔNACERSs_T2-T1_	0.57	6.94	-1.70	6.73	1.82	0.072
ΔNACERSs_T3-T1_	1.58	6.38	-0.30	8.12	1.43	0.160
ΔACERSs_T2-T1_	-2.30	6.89	2.23	7.42	-3.47	**0.001**
ΔACERSs_T3-T1_	-3.75	8.59	1.00	8.36	-3.07	**0.003**

Δ, Improvement.

FFMQ, Five Facet Mindfulness Questionnaire.

EI, Emotional Intelligence.

ERQ, Emotion Regulation Questionnaire; CR, Cognitive Reappraisal; ES, Expressive Suppression.

SDS, Self-Rating Depression Scale.

SAS, Self-Rating Anxiety Scale.

NACERSs, nonadaptive cognitive emotion regulation strategies; ACERSs, Adaptive cognitive emotion regulation strategies.

Bold values denote p<0.05.

### Mediation models

As there are only sustained training effects on FFMQ, EI and adaptive CERSs, the following analyses are based on these measurements.

Bivariate correlation analysis was conducted on the improvements of measurements at T2 and T3, as shown in **Table 3**. Generally, there are positive correlations among all the measurements. Therefore, we further test the mediation roles of adaptive CERSs and EI on the training effect of trait mindfulness by mediation effect analysis.

**Table 3:** Bivariate correlations between the improvements of measurements (*N*=120).

Measurements	2	3	4	5	6	7
1. Group	0.24^**^	0.19^*^	0.20^*^	0.23^*^	0.30^**^	0.27^**^
2. ΔFFMQ_T2-T1_	1	0.61^***^	0.36^***^	0.31^**^	0.27^**^	0.21^*^
3. ΔFFMQ_T3-T1_		1	0.28^**^	0.50^***^	0.18^*^	0.30^**^
4. ΔEI_T2-T1_			1	0.57^***^	0.34^***^	0.38^***^
5. ΔEI_T3-T1_				1	0.24^**^	0.55^***^
6. ΔACERSs_T2-T1_					1	0.56^***^
7. ΔACERSs_T3-T1_						1

***, p<0.001; **p<0.01; *p<0.05.

Δ, Improvement.

FFMQ, Five Facet Mindfulness Questionnaire.

EI, Emotional Intelligence.

ACERSs, Adaptive Cognitive emotion regulation strategies.

**Figure 1:**
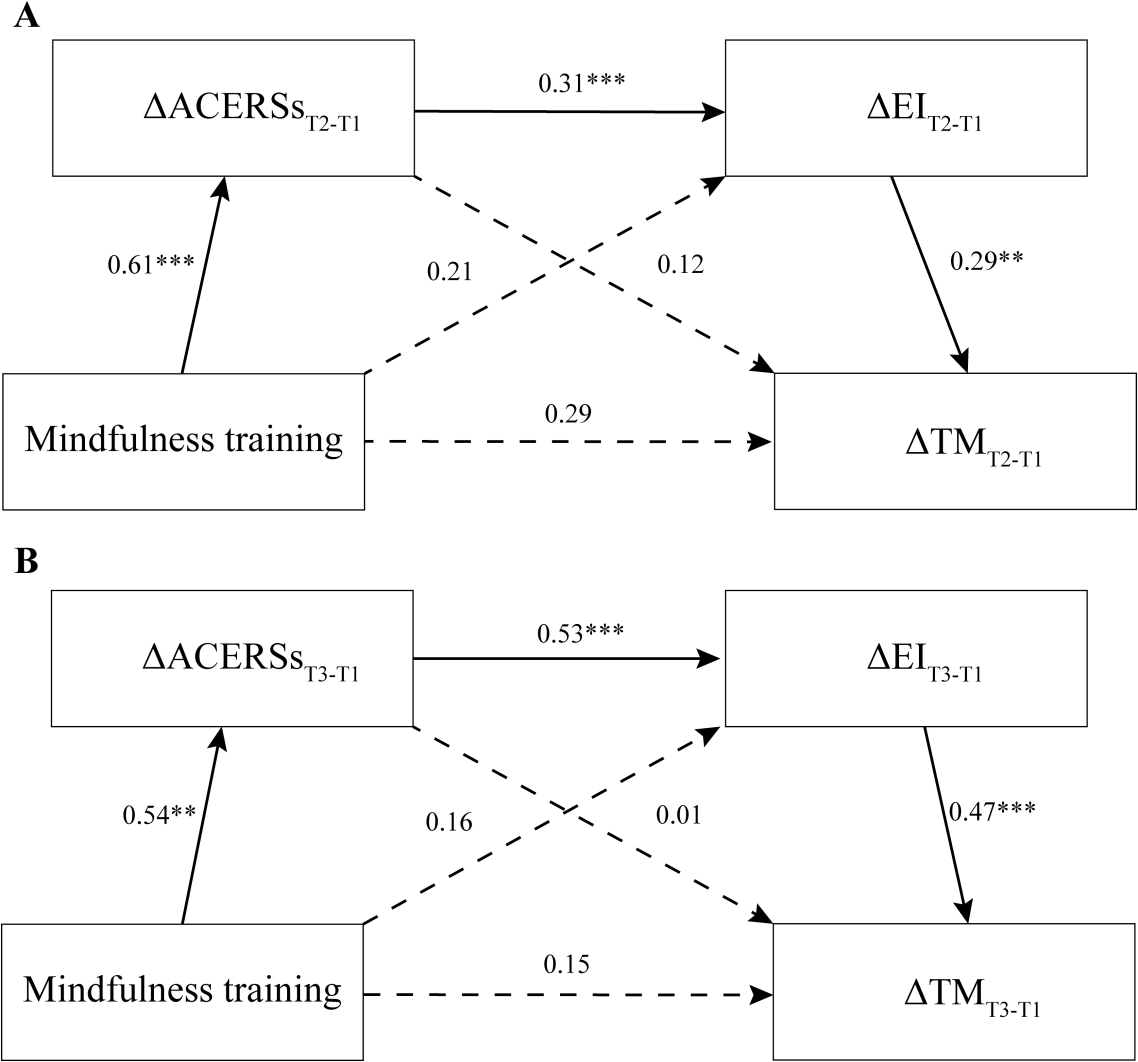
The sequential mediation effects of enhancements in adaptive cognitive emotion regulation strategies (ACERSs) and emotional intelligence (EI) in the relationship between mindfulness training and trait mindfulness (TM) at both T2 **(A)** and T3 **(B)** (Δ: Improvement; ***, p<0.001; **p<0.01).

First, cross-sectional mediation models were constructed, with mindfulness training (0=control group, 1=mindfulness group) serving as the independent variable, the improvements of adaptive CERSs and EI at T2 or T3 as mediators, and the improvement of FFMQ at T2 or T3 as the dependent variable (**Figure 1**). The modeling results at T2 and T3 by the PROCESS macro for SPSS (Model 6) showed that, there were significant total effects (bootstrap test with 2000 samples, T2: *β*=0.48, *t*=2.72, *p*=0.008; T3: *β*=0.38, *t*=2.08, *p*=0.039), but the direct effects were nonsignificant (T2: *β*=0.29, *t*=1.65, *p*=0.101; T3: *β*=0.15, *t*=0.93, *p*=0.355). Importantly, the sequential mediation effects were significant (T2: bootstrap95%CI [0.01, 0.13]; T3: bootstrap95%CI [0.04, 0.26]), indicating that enhancements in adaptive CERSs and EI could fully and sequentially mediate the impact of mindfulness training on trait mindfulness at both T2 and T3. Moreover, the separate mediating effects of the improvements of adaptive CERSs (T2: bootstrap95%CI [-0.05, 0.19]; T3: bootstrap95%CI [-0.09, 0.11]) and EI (T2: bootstrap95%CI [-0.04, 0.18]; T3: bootstrap95%CI [-0.07, 0.21]) were nonsignificant.

**Figure 2:**
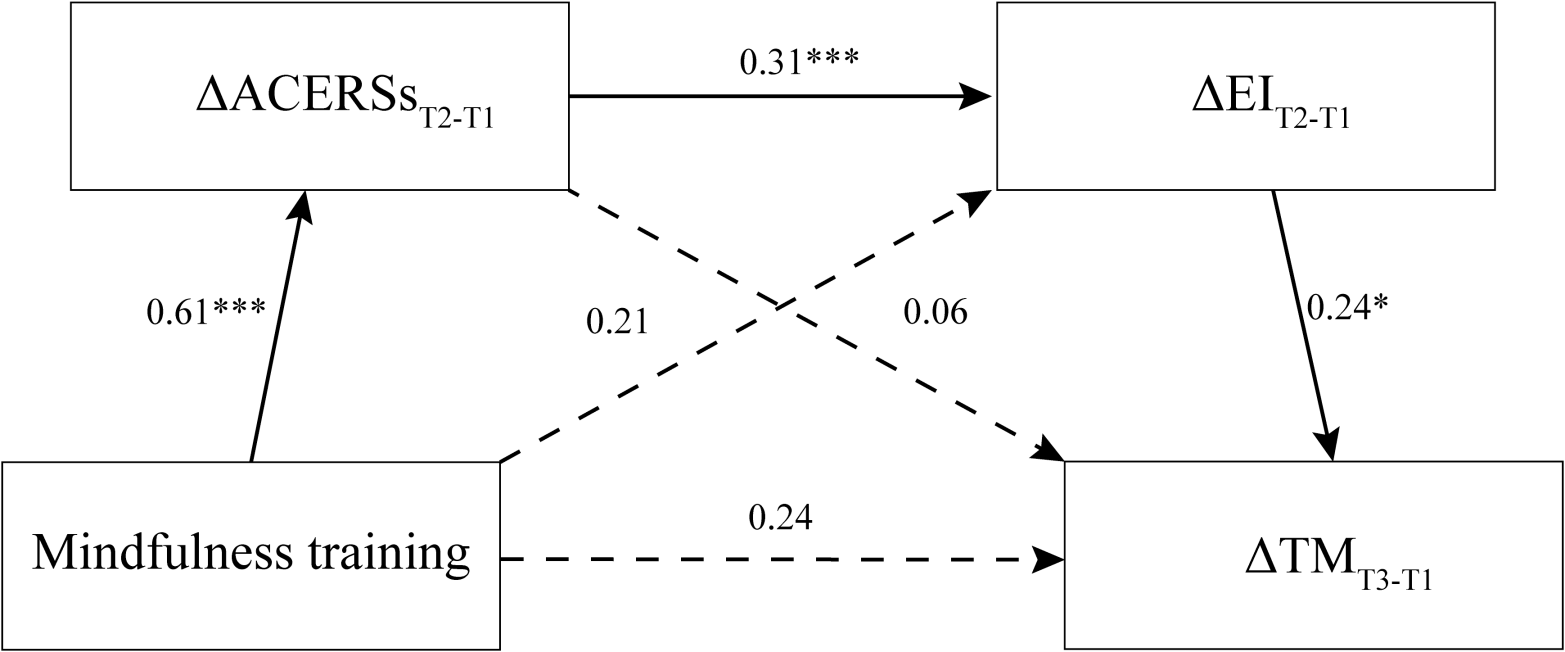
The longitudinal mediation effect of enhancements in adaptive cognitive emotion regulation strategies (ACERSs) and emotional intelligence (EI) at T2 in the relationship between mindfulness training and enhancements in trait mindfulness (TM) at T3 (Δ: Improvement; ***, p<0.001; *p<0.05).

Then, cross-lagged mediation models were constructed, with mindfulness training (0=control group, 1=mindfulness group) serving as the independent variable, the improvements of adaptive CERSs and EI at T2 as mediators, and the improvement of FFMQ at T3 as the dependent variable (**Figure 2**). The modeling results by the PROCESS macro for SPSS (Model 6) showed that, there were significant total effects (bootstrap test with 2000 samples, *β*=0.38, *t*=2.08, *p*=0.039), but the direct effects were nonsignificant (*β*=0.24, *t*=1.31, *p*=0.192). Importantly, the sequential mediation effect was still significant (bootstrap95%CI [0.004, 0.12]), indicating that enhancements in adaptive CERSs and EI at T2 could fully and sequentially mediate the impact of mindfulness training on trait mindfulness at T3. Meanwhile, the separate mediating effects of the improvements of both adaptive CERSs (bootstrap95%CI [-0.10, 0.16]) and EI (bootstrap95%CI [-0.04, 0.16]) were nonsignificant.

### Latent profile analysis for mindfulness group

In the present study, we did not find significant training effects on emotional symptoms, which may be inconsistent with our hypothesis. We conducted Latent Profile Analysis (LPA) using Mplus 8.3 to classify the members in the mindfulness group into subgroups.

Given the exploratory nature of LPA where the optimal number of latent classes remains undetermined *a priori*, it is necessary to systematically compare and evaluate competing models with progressively increasing class numbers. This evaluation should integrate statistical criteria, theoretical assumptions, sample size, as well as the interpretability and uniqueness of the classes (92, 93). Thus, we calculated the model fitness from 1 class to 6 classes based on the eight variables at baseline. Critical model fit evaluation information for these class solutions is presented in **Table 4**. The results revealed consistent declines in AIC, aBIC, and LMR-LRT values alongside uniformly significant BLRT p-values (<0.001), collectively failing to conclusively identify an optimal solution. The LMR-LRT for the two-class model, and the BIC and Entropy for the three-class model suggested good model fit. Considering sample distribution, we opted for the two-class solution due to its balanced distribution (Class 1: n=30; Class 2: n=30), contrasting with the three-class model (Class 1: n=26; Class 2: n=27; Class 3: n=7). Overall, the two-class solution effectively categorized participants in the mindfulness group, with the average latent class probabilities for the most likely latent class membership being 0.99 for Class 1 and 0.96 for Class 2.

**Table 4:** Model fit evaluation information.

Number of classes	AIC	BIC	aBIC	LMR-LRT		BLRT (p)	Entropy
				Value	p		
1-Class	3399.64	3433.15	3382.83				1.000
2-Class	3321.14	3373.49	3294.87	93.952	0.0636	<0.001	0.907
3-Class	3258.29	3329.49	3222.56	78.71	0.2361	<0.001	0.914
4-Class	3243.54	3333.59	3198.35	31.89	0.2311	<0.001	0.906
5-Class	3227.49	3336.39	3172.85	33.14	0.4207	<0.001	0.946
6-Class	3220.69	3348.45	3156.59	24.14	0.1119	<0.001	0.954

AIC, Akaike Information Criterion; BIC, Bayesian Information Criterion; aBIC, Sample-Size Adjusted BIC; LMR-LRT, Lo-Mendell-Rubin adjusted likelihood ratio test; BLRT, bootstrap likelihood ratio test.

Subsequently, independent-sample t-tests on the baseline measurements were conducted, based on the two-class grouping. As illustrated in **Table 5**, participants in Class 1 demonstrated significantly higher trait mindfulness and EI, coupled with lower expressive suppression, depression, anxiety, and nonadaptive CERSs. Thus, the first class could be named as the low-risk group, while the second class could be named as the high-risk group.

**Table 5:** Differences across the two classes in terms of the eight variables at baseline: descriptive statistics and results of independent-sample t-test (*N*=60).

Measurements	Class1 (Low-risk group; *n*=30)		Class2 (High-risk group; *n*=30)		t	p
	*M*	*SD*	*M*	*SD*		
FFMQ	90.73	8.66	82.37	9.47	3.57	**0.001**
EI	129.20	11.70	123.00	12.19	2.01	**0.049**
ERQ_CR	33.50	4.67	31.50	5.08	1.59	0.118
ERQ_ES	15.00	3.86	19.07	4.46	-3.78	**<0.001**
SDS	41.29	6.04	56.79	5.94	-10.02	**<0.001**
SAS	33.00	4.17	47.96	7.28	-9.76	**<0.001**
NACERSs	43.83	7.03	52.93	6.16	-0.533	**<0.001**
ACERSs	69.57	7.41	73.43	10.59	-1.64	0.107

FFMQ, Five Facet Mindfulness Questionnaire.

EI, Emotional Intelligence.

ERQ, Emotion Regulation Questionnaire; CR, Cognitive Reappraisal; ES, Expressive Suppression.

SDS, Self-Rating Depression Scale.

SAS, Self-Rating Anxiety Scale.

NACERSs, Nonadaptive cognitive emotion regulation strategies; ACERSs, Adaptive cognitive emotion regulation strategies.

Bold values denote p<0.05.

Then, independent-sample t-tests were conducted on the improvements of measurements at T2 and T3. As illustrated in **Table 6**, participants in Class 2 exhibited significant or marginally significant reductions in depression and anxiety at both T2 and T3 relative to Class 1. It indicates mindfulness training’s targeted efficacy specifically within high-risk populations such as anxious and depressive individuals. It should also be noted that there is no significant difference in improvements in most of the emotional regulations between groups, indicating similar sensitivity to mindfulness training on emotional regulation for the two subgroups.

**Table 6:** Differences across the two classes in terms of the improvements of eight measurements at T2 and T3: descriptive statistics and results of independent-sample t-test (*N*=60).

Measurements	Class1 (Low-risk group; *n*=30)		Class2 (High-risk group; *n*=30)		t	p
	*M*	*SD*	*M*	*SD*		
ΔFFMQ_T2-T1_	3.43	9.36	2.73	10.65	0.27	0.788
ΔFFMQ_T3-T1_	2.23	6.69	2.57	8.44	-0.17	0.866
ΔEI_T2-T1_	1.67	6.65	2.67	12.01	-0.40	0.691
ΔEI_T3-T1_	0.03	6.75	0.77	10.58	-0.32	0.750
ΔERQ_CR_T2-T1_	0.43	4.52	0.80	4.65	-0.31	0.758
ΔERQ_CR_T3-T1_	0.30	4.64	1.10	4.94	-0.65	0.520
ΔERQ_ES_T2-T1_	-0.10	4.13	-1.77	3.21	1.75	0.086
ΔERQ_ES_T3-T1_	1.93	3.89	-0.50	3.79	2.46	**0.017**
ΔSDS_T2-T1_	-1.46	7.25	-5.54	8.58	1.99	0.051
ΔSDS_T3-T1_	1.13	7.86	-4.13	9.44	2.34	**0.023**
ΔSAS_T2-T1_	0.83	5.71	-3.83	7.02	2.83	**0.006**
ΔSAS_T3-T1_	4.67	8.52	0.29	8.11	2.04	**0.046**
ΔNACERSs_T2-T1_	-0.70	6.68	-2.70	6.74	1.16	0.253
ΔNACERSs_T3-T1_	1.20	9.42	-1.80	6.38	1.44	0.154
ΔACERSs_T2-T1_	2.73	6.83	1.73	8.05	0.32	0.606
ΔACERSs_T3-T1_	2.70	7.65	-0.70	8.82	1.59	0.116

Δ, Improvement.

FFMQ, Five Facet Mindfulness Questionnaire.

EI, Emotional Intelligence.

ERQ, Emotion Regulation Questionnaire; CR, Cognitive Reappraisal; ES, Expressive Suppression.

SDS, Self-Rating Depression Scale.

SAS, Self-Rating Anxiety Scale.

NACERSs, Nonadaptive cognitive emotion regulation strategies; ACERSs, Adaptive cognitive emotion regulation strategies.

Bold values denote p<0.05.

## Discussion

The present study, utilizing a randomized controlled trial design with 120 participants, initially revealed that a 5-week EmotionCore mindfulness training significantly improved adaptive CERSs, EI, and trait mindfulness, with effects lasting at least one month post-training. Furthermore, both cross-section and longitudinal mediation analysis revealed that the improvements in adaptive CERSs and EI completely and sequentially mediated the relationship between mindfulness training and the enhancement of trait mindfulness. Lastly, when implementing LPA, the high-risk group with high levels of anxiety and depression at baseline demonstrated greater reductions in anxiety and depression compared to the low-risk group, suggesting differential intervention effectiveness based on initial symptom severity.

In our study, we revealed long-lasting training effects of the innovative 5-week EmotionCore mindfulness program on multiple measurements, including adaptive CERSs, EI, and trait mindfulness. These findings are consistent with previous studies showing that mindfulness training improved the utilization of adaptive CERSs (49, 94, 95), enhanced EI (71–73, 96–99), and fortified trait mindfulness (100–106). However, no long-lasting improvements in nonadaptive CERSs, expressive suppression and cognitive reappraisal were found in the present study. It may be partly inconsistent with previous studies demonstrating that mindfulness training reduces the utilization of nonadaptive CERSs (49, 94, 95) and improves rumination, catastrophizing, cognitive reappraisal, and expressive suppression (50–52, 107–110). Notably, our program led to enhanced adaptive CERSs. The differential outcomes for cognitive appraisal and adaptive CERSs in the present study may reflect differences in conceptual structures stemming from measurement tool variances. Cognitive reappraisal is defined as a form of cognitive change that involves construing a potentially emotion-eliciting situation in a way that changes its emotional impact (111), while adaptive CERSs refers to thoughts of attaching a positive meaning to the event in terms of personal growth (44). By comparison, adaptive CERSs emphasize optimistic reinterpretation over mere cognitive shifts. It appears that the EmotionCore mindfulness training is more effective in cultivating positive cognitive reframing. The findings reinforce theoretical frameworks positing mindfulness-driven cognitive shifts through decentering mechanisms (34–37, 112), which encourages positive psychological outcomes. Nevertheless, it is worth investigating if there are other factors influencing the training effect on cognitive reappraisal and expressive suppression.

Interestingly, both cross-section and longitudinal mediation models revealed that adaptive CERSs and EI are the mediating pathways through which EmotionCore mindfulness training exerts its effects on trait mindfulness. It appears that the 5-week EmotionCore mindfulness training initially changes adaptive CERSs, and then enhances EI, indicating the developmental pathway that cognition shift in emotion experiences precede, thereby facilitating the growth of emotional competencies. These findings support the process model of emotion regulation, specifying the sequence of steps involved in emotion generation (113, 114). The first step is situation selection and modification. Selection refers to taking actions that make it more (or less) likely that one will be in an expected situation that will give rise to desirable (or undesirable) emotions, while modification refers to taking actions that directly alter a situation in order to change its emotional impact. The second step is attentional deployment, which refers to directing one’s attention with the goal of influencing one’s emotional response. The third step is cognitive change, which refers to modifying one’s appraisal of a situation in order to alter its emotional impact. The fourth step is response modulation, which refers to directly influencing experiential, behavioral, or physiological components of the emotional response after the emotion is well developed. Previous studies have revealed that EI is mainly correlated with positive reappraisal (115), and positive refocusing (116). Other studies showed that adaptive CERSs positively predicted EI (116) and CERS predicted trait EI (67), consistent with the present study. Therefore, it is plausible that only by learning and flexibly applying emotion regulation strategies can one enhance their emotion regulation ability, a component of EI.

Sequentially, the improvement of EI led to enhanced trait mindfulness. According to the four-component model of mindfulness, four components are proposed to describe the mechanism through which mindfulness training exerts its effects: 1) attention regulation, 2) body awareness, 3) emotion regulation, and 4) change in perspective on the self (117). When emotional reactions are triggered, the executive attention system could detect conflict with mindful state maintenance and physiological aspects of feelings, enabling accurate emotional response identification. Emotion regulation processes then engage to replace habitual reactions, which facilitates response prevention through nonreactivity, leading to extinction/reconsolidation. This allows individuals to experience the transient nature of perceptions/emotions/cognitions rather than habitual reactions. Awareness of this transience alters the perspective on the self, which refers to a change in perspective about the sense of self and an alteration in first-person subjective experience, described as observer perspective. Evidence has exhibited that emotion regulation, body awareness, and change of self appeared to be the most important mechanisms of action through which mindfulness exerts its beneficial effects on mental health (118, 119). Therefore, after mindfulness training improves adaptive CRESs and EI, the perspective on the self is changed, correlated with changes in trait mindfulness. Based on the present and previous findings, we propose that the EmotionCore mindfulness training may initiate its effects on the utilization of emotion regulation strategies through enhanced meta-awareness, subsequently fostering improvements in EI. Crucially, EI enhancement may then reciprocally strengthen trait mindfulness, thereby establishing a positive feedback loop. Further, the full mediation effect seems to indicate that mindful emotion training cannot directly improve trait mindfulness.

Surprisingly, we did not find long-lasting training effects of the 5-week EmotionCore mindfulness training on anxiety or depression in the first place. This is unusual as numerous previous studies have shown strong evidence that mindfulness training is an effective intervention for emotional disorders (12, 16–23, 15). Therefore, it is quite necessary to account for the negative result of the emotional symptoms. We note that the effectiveness of mindfulness training may be different across different populations. Meta-analyses revealed that among healthy individuals, healthcare professionals benefited most from mindfulness training, followed by general populations and then students (120). Studies showed lower benefits observed in student populations compared to general populations or working adults (121), and the intervention effectiveness of mindfulness training for clinical samples was better than that for healthy individuals in Eastern countries (122). In the present study, the participants are healthy undergraduates who have relatively low depressive and anxious levels, and exhibit relatively lower sensitivity to mindfulness training in previous studies. The floor effect may prevent further reductions in emotional symptoms. The present LPA results revealed that the training efficacy of EmotionCore mindfulness training for both anxiety and depression was moderated by the levels of emotional health at baseline. The high-risk group, characterized by higher levels of anxiety and depression at baseline, exhibited symptom reduction at both T2 and T3 follow-ups compared to their low-risk counterparts. This pattern resonates with the “floor effect” hypothesis in psychotherapeutic interventions (123). These findings highlight the EmotionCore mindfulness training program’s targeted therapeutic potency for the emotional symptoms among high-risk populations and its general efficacy for enhancing emotional regulation.

## Limitations

First, the study focused solely on undergraduate populations, which may limit the generalization of its conclusions. Future research should investigate the efficacy of EmotionCore mindfulness training and its mechanisms across diverse groups, including clinical and cross-cultural samples.

Second, key variables such as emotion regulation relied on self-report measures, making them susceptible to response biases (e.g., social desirability) and inadequate for capturing objective behavioral or physiological indicators. For example, heart rate variability is closely associated with emotion regulation (124, 125); amygdala–prefrontal cortex connectivity is closely associated with mindfulness (126–129). Subsequent studies should incorporate multimodal assessments such as experimental emotion-regulation paradigms and neuroimaging to further verify the impact of EmotionCore mindfulness training on emotion regulation and its underlying neural mechanisms.

Third, the current research merely assessed the sustained effects of EmotionCore training at a one-month follow-up. In the future, conducting longitudinal studies is imperative to thoroughly explore the sustained efficacy of this training through extended follow-up periods.

Fourth, the latent profile analysis primarily focused on baseline emotional problems, emotion regulation strategies, emotional intelligence, and trait mindfulness, overlooking potential moderators like personality traits and environmental factors. Future research should identify supplementary moderators to enhance personalized approaches for high-risk subgroups while establishing a hierarchical intervention framework that tailors training programs to participants’ distinct psychological profiles.

Fifth, the lack of control over participants’ spontaneous mindfulness practice during the follow-up period introduces a limitation. While we could not restrict such practices (due to ethical considerations), their potential influence on T3 outcomes should be acknowledged. Evidence revealed that the majority of participants receiving an 8-week mindfulness training demonstrated ongoing compliance with the mindfulness practice at 3 years (130). Future research may explore the role of habitual practice in the long-term effects.

Lastly, the waitlist control group was administered two introductory lectures on mindfulness concepts in this study, whereas the training group participated in a 5-week systematic mindfulness intervention. Although preliminary analyses revealed no significant between-group differences in key baseline variables, someone may argue that the difference in improvements between groups may stem from factors other than mindfulness. A meta-analysis study suggested that active (including guided breathing, health education, lifestyle education, math, nutrition classes, reading groups, relaxation, or sitting in silence) and passive (including simple test-retest or waitlist controls) control groups produced statistically indistinguishable results, indicating that mindfulness is a crucial ingredient in producing the training effects (131). Nevertheless, future studies could use an active control group to more rigorously control non-specific effects.

## Conclusions

This study provides robust evidence for the efficacy of a 5-week EmotionCore mindfulness training program in immediately and sustainably enhancing adaptive CERSs, EI, and trait mindfulness among undergraduates. Critically, the findings elucidate the full and sequential mediating role of adaptive CERSs and EI in linking mindfulness training to trait mindfulness, supporting a developmental pathway in that cognitive regulatory capacities precede emotional competencies. Notably, the training demonstrated targeted benefits for high-risk individuals with elevated baseline anxiety and depressive symptoms, who exhibited greater reductions in anxiety and depression compared to the low-risk group.

## Data Availability

The raw data supporting the conclusions of this article will be made available by the authors, without undue reservation.
