# Supplementary material for: The sustained effect of 5-week EmotionCore mindfulness training on emotion regulation and emotional intelligence: heterogeneous benefits for depression and anxiety across subgroups
Source: Front Psychiatry. 2025 Jul 18;16:1622626. doi: 10.3389/fpsyt.2025.1622626 (PMC12313724; doi:10.3389/fpsyt.2025.1622626)
Supplement: Supplementary file 1 [file Table1.docx]

**Table S1**  Results of descriptive statistics and MANCOVAs at T1 (*N*=120)

| Scales | Subscales | Control group  (*n*=60) | |  | Mindfulness group  (*n*=60) | | *F* | *p* | $\eta_{p}^{2}$ |
| --- | --- | --- | --- | --- | --- | --- | --- | --- | --- |
|  |  | *M* | *SD* |  | *M* | *SD* |  |  |  |
| FFMQ | Observing | 20.80 | 3.87 |  | 20.97 | 4.01 | 0.05 | 0.817 | <0.001 |
|  | Describing | 19.08 | 3.98 |  | 17.85 | 4.64 | 2.44 | 0.121 | 0.02 |
|  | Acting with awareness | 14.85 | 3.69 |  | 15.43 | 3.52 | 0.79 | 0.377 | 0.01 |
|  | Non-judging | 13.70 | 4.06 |  | 14.27 | 3.90 | 0.61 | 0.437 | 0.01 |
|  | Non-reacting | 18.78 | 2.91 |  | 18.03 | 3.84 | 1.46 | 0.230 | 0.01 |
|  | Total | 87.22 | 7.54 |  | 86.55 | 9.94 | 0.17 | 0.680 | 0.001 |
| EIS | AEOE | 44.32 | 4.48 |  | 45.10 | 6.03 | 0.65 | 0.421 | 0.01 |
|  | RE | 32.35 | 3.24 |  | 31.47 | 3.40 | 2.12 | 0.148 | 0.02 |
|  | AOE | 24.35 | 2.56 |  | 24.03 | 2.67 | 0.44 | 0.509 | 0.004 |
|  | UE | 26.07 | 2.46 |  | 25.50 | 2.50 | 1.57 | 0.213 | 0.01 |
|  | Total | 127.08 | 10.12 |  | 126.10 | 12.25 | 0.23 | 0.633 | 0.002 |
| ERQ | CR | 32.65 | 4.23 |  | 32.50 | 4.95 | 0.03 | 0.859 | <0.01 |
|  | ES | 17.87 | 4.18 |  | 17.03 | 4.62 | 1.08 | 0.302 | 0.01 |
| CERSs | Self-blame | 12.98 | 2.39 |  | 12.63 | 2.28 | 0.68 | 0.413 | 0.01 |
|  | Acceptance | 14.48 | 2.06 |  | 13.93 | 2.83 | 1.48 | 0.227 | 0.01 |
|  | Rumination | 14.47 | 2.82 |  | 14.25 | 3.31 | 0.15 | 0.700 | 0.001 |
|  | Positive refocusing | 14.75 | 3.14 |  | 13.80 | 2.80 | 3.06 | 0.083 | 0.03 |
|  | Refocus on planning | 15.83 | 2.91 |  | 15.28 | 2.71 | 1.15 | 0.286 | 0.01 |
|  | Positive reappraisal | 16.07 | 2.56 |  | 15.80 | 3.07 | 0.27 | 0.607 | 0.002 |
|  | Putting into perspective | 12.83 | 2.75 |  | 12.68 | 2.85 | 0.09 | 0.770 | 0.001 |
|  | Catastrophizing | 9.62 | 3.59 |  | 9.57 | 3.30 | 0.01 | 0.937 | <0.001 |
|  | Blaming others | 11.02 | 3.19 |  | 11.93 | 2.34 | 3.22 | 0.075 | 0.03 |
|  | Non-adaptive | 48.08 | 7.70 |  | 48.38 | 8.00 | 0.04 | 0.835 | <0.001 |
|  | Adaptive | 73.97 | 9.27 |  | 71.50 | 9.27 | 2.12 | 0.148 | 0.02 |
| SDS |  | 47.75 | 10.13 |  | 49.04 | 9.82 | 0.50 | 0.479 | 0.004 |
| SAS |  | 42.46 | 9.60 |  | 40.48 | 9.57 | 1.28 | 0.260 | 0.01 |

FFMQ: Five Facet Mindfulness Questionnaire.

EIS: Emotional intelligence Scale; AEOE: appraisal and expression of own emotion; RE: regulation of emotion; AOE: appraisal of other’s emotion; UE: utilization of emotion.

ERQ: Emotion Regulation Questionnaire; CR: Cognitive Reappraisal; ES: Expressive Suppression.

SDS: Self-Rating Depression Scale.

SAS: Self-Rating Anxiety Scale.

CERSs: Cognitive emotion regulation strategies.

**Table S2**  Results of descriptive statistics and MANCOVAs at T2 and T3 (*N*=120)

| Time points | Scales | Subscales | Control group  (*n*=60) | |  | Mindfulness group  (*n*=60) | | *F* | *p* | $\eta_{p}^{2}$ |
| --- | --- | --- | --- | --- | --- | --- | --- | --- | --- | --- |
|  |  |  | *M* | *SD* |  | *M* | *SD* |  |  |  |
| T2 | FFMQ | Observing | 20.55 | 3.59 |  | 22.35 | 3.64 | 7.44 | **.007** | 0.06 |
|  |  | Describing | 19.08 | 3.76 |  | 18.73 | 4.11 | 0.24 | .627 | <0.01 |
|  |  | Acting with awareness | 14.00 | 3.62 |  | 14.22 | 3.81 | 0.10 | .750 | <0.01 |
|  |  | Non-judging | 13.57 | 3.38 |  | 14.90 | 4.43 | 3.44 | **.066** | 0.03 |
|  |  | Non-reacting | 19.02 | 3.06 |  | 19.43 | 3.12 | 0.54 | .462 | <0.01 |
|  |  | Total | 86.22 | 7.28 |  | 89.63 | 10.68 | 4.19 | **.043** | 0.03 |
|  | EI | AEOE | 44.32 | 4.22 |  | 46.07 | 5.92 | 3.48 | **.065** | 0.03 |
|  |  | RE | 31.45 | 3.45 |  | 32.08 | 3.48 | 1.00 | .319 | 0.01 |
|  |  | AOE | 24.17 | 3.03 |  | 24.33 | 2.89 | 0.09 | .759 | <0.01 |
|  |  | UE | 25.23 | 2.60 |  | 25.78 | 2.58 | 1.35 | .248 | 0.01 |
|  |  | Total | 125.17 | 11.62 |  | 128.27 | 12.26 | 2.02 | .158 | 0.02 |
|  | ERQ | CR | 32.58 | 4.09 |  | 33.12 | 4.00 | 0.52 | .472 | 0.00 |
|  |  | ES | 18.68 | 3.64 |  | 16.10 | 4.28 | 12.69 | **.001** | 0.10 |
|  | CERSs | Self-blame | 12.85 | 1.99 |  | 12.20 | 2.46 | 2.53 | 0.114 | 0.02 |
|  |  | Acceptance | 14.22 | 1.98 |  | 14.37 | 2.57 | 0.13 | .721 | <0.01 |
|  |  | Rumination | 14.57 | 2.52 |  | 14.57 | 2.82 | 0.00 | 1.000 | 0.00 |
|  |  | Positive refocusing | 14.47 | 2.55 |  | 14.58 | 2.38 | 0.07 | .796 | <0.01 |
|  |  | Refocus on planning | 15.43 | 2.97 |  | 15.48 | 2.63 | 0.01 | .922 | <0.01 |
|  |  | Positive reappraisal | 15.42 | 2.65 |  | 16.47 | 2.54 | 4.91 | **.029** | 0.04 |
|  |  | Putting into perspective | 12.13 | 2.38 |  | 12.83 | 3.17 | 1.87 | .174 | 0.02 |
|  |  | Catastrophizing | 9.83 | 3.08 |  | 8.43 | 3.12 | 6.11 | **.015** | 0.05 |
|  |  | Blaming others | 11.40 | 2.57 |  | 11.48 | 3.00 | 0.03 | .871 | <0.01 |
|  |  | Non-adaptive | 48.65 | 6.36 |  | 46.68 | 7.13 | 2.54 | .114 | 0.02 |
|  |  | Adaptive | 71.67 | 8.83 |  | 73.73 | 9.16 | 1.58 | .211 | 0.01 |
|  | SDS |  | 46.40 | 8.68 |  | 45.54 | 9.91 | 0.25 | .617 | 0.00 |
|  | SAS |  | 43.65 | 9.65 |  | 38.98 | 8.08 | 8.25 | .005 | 0.07 |
| T3 | FFMQ | Observing | 20.80 | 3.60 |  | 21.67 | 3.78 | 1.65 | .201 | 0.01 |
|  |  | Describing | 19.88 | 3.25 |  | 19.53 | 4.25 | 0.26 | .613 | <0.01 |
|  |  | Acting with awareness | 14.02 | 3.61 |  | 14.33 | 3.94 | 0.21 | .647 | <0.01 |
|  |  | Non-judging | 12.62 | 3.39 |  | 13.88 | 4.28 | 3.23 | .075 | 0.03 |
|  |  | Non-reacting | 19.60 | 2.68 |  | 19.53 | 3.39 | 0.01 | .905 | <0.01 |
|  |  | Total | 86.92 | 6.33 |  | 88.95 | 9.82 | 1.82 | .180 | 0.02 |
|  | EI | AEOE | 44.13 | 4.38 |  | 45.97 | 5.63 | 3.96 | **.049** | 0.03 |
|  |  | RE | 31.08 | 3.64 |  | 31.65 | 3.25 | 0.81 | .370 | 0.01 |
|  |  | AOE | 23.58 | 2.75 |  | 23.88 | 2.82 | 0.35 | .556 | <0.01 |
|  |  | UE | 24.37 | 3.03 |  | 25.00 | 2.79 | 1.42 | .235 | 0.01 |
|  |  | Total | 123.17 | 12.01 |  | 126.50 | 12.61 | 2.20 | .141 | 0.02 |
|  | ERQ | CR | 32.52 | 4.63 |  | 33.20 | 4.66 | 0.65 | .422 | 0.01 |
|  |  | ES | 18.98 | 3.20 |  | 17.75 | 4.55 | 2.95 | .089 | 0.02 |
|  | CERSs | Self-blame | 12.60 | 2.18 |  | 12.25 | 2.61 | 0.63 | .427 | 0.01 |
|  |  | Acceptance | 13.25 | 2.53 |  | 14.20 | 2.59 | 4.13 | **.044** | 0.03 |
|  |  | Rumination | 13.72 | 2.62 |  | 14.38 | 3.10 | 1.62 | .206 | 0.01 |
|  |  | Positive refocusing | 14.12 | 2.66 |  | 13.78 | 2.55 | 0.49 | .484 | 0.00 |
|  |  | Refocus on planning | 14.85 | 2.77 |  | 15.32 | 2.38 | 0.98 | .324 | 0.01 |
|  |  | Positive reappraisal | 15.12 | 2.62 |  | 16.05 | 2.74 | 3.64 | **.059** | 0.03 |
|  |  | Putting into perspective | 12.88 | 2.69 |  | 13.15 | 2.39 | 0.33 | .568 | <0.01 |
|  |  | Catastrophizing | 11.05 | 3.21 |  | 9.72 | 3.08 | 5.39 | **.022** | 0.04 |
|  |  | Blaming others | 12.30 | 1.87 |  | 11.73 | 2.31 | 2.18 | .143 | 0.02 |
|  |  | Non-adaptive | 49.67 | 5.77 |  | 48.08 | 8.06 | 1.53 | .218 | 0.01 |
|  |  | Adaptive | 70.22 | 10.11 |  | 72.50 | 8.74 | 1.75 | .188 | 0.01 |
|  | SDS |  | 48.85 | 10.53 |  | 47.54 | 10.55 | 0.47 | .496 | 0.00 |
|  | SAS |  | 45.88 | 12.16 |  | 42.96 | 10.26 | 2.02 | .158 | 0.02 |

FFMQ: Five Facet Mindfulness Questionnaire.

EI: Emotional Intelligence; AEOE: appraisal and expression of own emotion; RE: regulation of emotion; AOE: appraisal of other’s emotion; UE: utilization of emotion.

ERQ: Emotion Regulation Questionnaire; CR: Cognitive Reappraisal; ES: Expressive Suppression.

SDS: Self-Rating Depression Scale.

SAS: Self-Rating Anxiety Scale.

CERSs: Cognitive emotion regulation strategies.
